# The application of electrophysiology in the correction of spastic torticollis by triple surgery—report of 96 cases

**DOI:** 10.1186/s12891-023-06518-3

**Published:** 2023-06-06

**Authors:** Jun Wu, Hao Xu, Lu Wang, Jun Li

**Affiliations:** Departmennt of Neurosurgery, Tongji Medical College, The Central Hospital of Wuhan, Huazhong University of Science and Technology, No. 26 Shengli Street, Jiang An District, Wuhan, Hubei 430014 China

**Keywords:** Spastic torticollis, Electrophysiological application, The ‘triple operation’

## Abstract

**Objective:**

To investigate the effect and value of electrophysiology in the ‘triple operation’ (selective excision of spastic muscles in the neck, selective resection of the posterior branch of the cervical nerve and accessory neurotomy) of spastic torticollis.

**Methods:**

Preoperative electromyography (EMG) examination was performed on 96 patients with spastic torticollis treated in our hospital from January 2015 to December 2019. The results were used to assess the responsible muscles’ primary or secondary position and the function of antagonistic muscles and to formulate a personalised surgical plan. A Cascade PRO 16-channel electrophysiological diagnostic system (produced by Cadwell, USA) was used to record the evoked EMG. Target muscles were denervated under intraoperative electrophysiological monitoring and re-examined by EMG six months later to evaluate the efficacy.

**Results:**

The satisfactory rate of target muscle denervation was 95%, and the overall good rate was 79.1%.

**Conclusion:**

Electrophysiological examination and intraoperative application may have a positive value in the selection of the operative method, improving the rate of denervation and evaluating the prognosis of the ‘triple operation’.

## Introduction

Spastic torticollis (ST) is a dystonia disease mainly occurring in the neck muscles. It produces distorted, skewed and postural abnormalities in the head and neck due to intermittent or continuous involuntary contraction of the neck muscles [1]. The aetiology of ST is still inconclusive, and some researchers believe that primary ST has genetic locus abnormalities at the DYT6 and DYT7 loci [2]. Drugs have limited effects on ST, although kreotoxin is an effective non-surgical treatment. Still, its efficacy is short, repeated injections are required and long-term injections are prone to resistance [3]. Surgery is a common treatment for ST, and ‘triple surgery’ has a good therapeutic effect on ST [4]. Electromyography (EMG) is a means to perform an auxiliary examination of diseases. After this examination, the functional status of peripheral nerves, neurons, neuromuscular joints and muscles can be determined. An EMG of patients with ST during the perioperative period can reduce the degree of muscle and nerve damage and help protect the patients’ neck function. It not only improves surgical efficacy but also improves prognosis [5].

It was known that EMG was helpful in improving the efficacy of triple surgery. Therefore, we did not focus on the differences in results compared between groups, but rather on the operation and experience of EMG in practical applications. This will be a supplement to existing research on the application of EMG in triple surgery. This study conducted a combination of preoperative electromyogram evaluation and intraoperative guidance, evaluated the role of EMG in ‘triple operation’ and provided data support for improving surgical outcomes.

## Information and methods

### General information

Ninety-six patients with ST who were treated in our hospital from 2015 to 2019 were selected to participate in this study. After admission, cervical computerised tomography or magnetic resonance imaging examinations were routinely performed to exclude cervical diseases and to determine the hypertrophy of neck muscles due to spasm. Then, an EMG examination was performed. Inclusion criteria: 1) the patient was older than 18 years old, regardless of gender; 2) the patient had not received surgical treatment before participating in this study; 3) the patient was diagnosed with spastic torticollis; 4) the patient underwent a ‘triple operation’ for spastic torticollis and 5) the patient voluntarily participated in this study and signed an informed consent form. Exclusion criteria: (1) the patient had ST combined with other cervical spondylosis or (2) the patient was a pregnant woman. This study was approved by the ethics committee of the hospital [No: Y-SWJW-L2021(57)], and all patients signed the informed consent.

### Preoperative EMG examination

The chief physician of the research team developed an EMG method based on patients’ symptoms and spastic types and identified the muscle groups that required EMG (such as the contralateral sternocleidomastoid muscle and contralateral trapezius muscle). The EMG was conducted by two physicians from the research team with more than ten years of experience performing EMGs. The Key point work electrophysiology instrument was used to puncture and perform recordings in sequence according to the body surface projection of muscle anatomy. Generally, the recording started from the midline and proceeded to both sides; puncture points of the deep neck muscles were chosen from the areas with less muscle coverage. Another point was also selected, first shallow and then deep, and the cervical muscles with mutual coverage were recorded. The EMG signals of muscles during involuntary and voluntary movements were recorded to assess muscle spasm and function. Two physicians jointly confirmed the result of the EMG. If there was disagreement, the two physicians and the chief physician discussed the issue and reached a concensus.

### Analysis of EMG results

#### Responsible and antagonistic muscles

According to the different roles played by the neck muscles in the attack of ST, the measured muscles were divided into responsible and antagonistic muscles. The distribution of muscles was as follows: ① The responsible muscles of rotational type ST included the splenius cervicis, semispinalis, levator scapulae, scalene, longissimus capitis and contralateral sternocleidomastoid muscle on the rotating side at the lateral neck; the antagonist muscle was the contralateral homonymous muscle. ② The responsible muscles of lateral bending type ST included the sternocleidomastoid, splenius cervicis, semispinalis, levator scapulae, scalene and longissimus capitis on the flexion side; the antagonist muscle was the contralateral homonymous muscle. ③ The responsible muscles of retroflexion type ST included bilateral splenius cervicis, semispinalis, rectus capitis posterior major, superior oblique muscle, inferior oblique muscle and longissimus capitis; the antagonist muscle was the bilateral sternocleidomastoid. ④ The responsible muscles of retroflexion type ST included the bilateral sternocleidomastoid; the antagonist muscles were the muscles associated with hypokinesis [6].

#### Spastic muscle classification

The degree of muscle spasm was distinguished according to the EMG recording results, and the spastic muscles were divided into three grades. Grade III spastic muscles: presenting a complete interference pattern, with a large number of motor units that could be recorded, amplitude > 1000 µV and frequency 30–50 Hz; Grade II spastic muscles: part of motor units involved in the movement, electrical activity weaker than the interference wave can be recorded in the form of a reduced or incomplete interference pattern, amplitude 400–1000 µV and frequency 10–30 Hz. Grade I spastic muscles: single motor unit action potentials appearing intermittently in series, with amplitude < 400 µV and frequency 5–10 Hz [7]. The product of EMG amplitude and frequency could also be used as the EMG index. The EMG index of spastic muscles was recorded when moving involuntarily and when contracting hard against external forces during voluntary movements. The differences between them were compared: a difference of ≤ 10% meant that the spastic muscle contraction was close to the maximum contraction force of the muscle, which was a Grade III spasm; a difference of ≥ 80% meant that the spastic muscle only used a small part of its force to participate in the muscle spasm, which was a Grade I spasm; in between, the spastic muscle was a Grade II spasm. The EMG index also assessed antagonist muscle function and the degree of intraoperative denervation.

#### Functional assessment of antagonistic muscles

The function of the antagonist muscle was evaluated by EMG and classified as excellent, good or poor. The EMG activity of the antagonist muscle was measured during involuntary movements and when the muscle contracted hard to correct the abnormal head position. The EMG index was calculated. If the difference between the two EMG indexes was ≥ 80%, and the abnormal head position could be corrected at the same time, they were termed excellent; if the difference between the EMG indexes was ≤ 10% or completely electrically resting, and the abnormal head position could not be improved at all, they was termed poor; those in between, in which the abnormal head position could be partially corrected, were termed good. Antagonist muscles with significant spasm before the surgery were also termed poor for their inability to improve abnormal head position at near maximum EMG activity [8].

### Surgical method selection

According to the preoperative assessment of the responsible muscle, the following ‘triple operation’ was adopted: neurectomy and/or myotomy for Grade III spasm, neurectomy for Grade II spasm, and partial neurectomy or no treatment for Grade I spasm. The following surgical methods were included: ① Accessory nerve transection: denervation of the sternocleidomastoid branch and denervation or preservation of the trapezius branch depending on the spasticity of the trapezius branch. ② Selective muscle resection: partial resection or dissection of the muscles that played a major role in the responsible muscles and were difficult to denervate, such as the splenius capitis and/or scalenus muscle. ③ Selective resection of the posterior branch of the cervical nerve 1–6 (C1–C6): selective resection of the posterior branch of the cervical nerve 1–6 (C1–C6) to achieve denervation of the posterior cervical responsible muscles.

For different types of torticollis, different surgical combinations were adopted: (1) Rotation type: ① plus ② plus ③, i.e. contralateral accessory nerve dissection plus partial excision of the posterior cervical muscle on the rotating side plus selective resection of the posterior branches of the cervical nerve 1–6 (C1–C6); (2) Lateral bending type: ① plus ② plus ③, i.e. accessory nerve dissection on the bending side plus partial resection of the posterior cervical muscle on the same side plus selective resection of the posterior branches of the cervical nerve 1–6 (C1–C6); (3) Anterior flexion type: ①, i.e. bilateral accessory nerve dissection; (4) Retroflexion type: ② plus ③, i.e. bilateral partial resection of posterior cervical muscles plus selective dissection of posterior branches of cervical nerves 1–6 (C1–C6).

### Intraoperative electrophysiological monitoring

A Cascade PRO 16-channel electrophysiological diagnostic system produced by Cadwell, USA, was used to record the evoked EMG. The recording electrode was inserted into one end of the target muscle. After separating the nerve trunk and branches, 0.5–2 mA stimulation was given with the stimulating electrode to evoke EMG activity. The strength of the recorded EMG activity was used to clarify the innervation, determine its effect on muscle spasm and calculate the EMG index before denervation based on the maximum EMG activity of the target muscle. The trunk and branches that evoked significant EMG activity were dissected. After denervation, the target muscle was probed again with the stimulating electrode. An 80% decrease in the recorded EMG index was used as a satisfactory criterion for denervation. The innervated nerve or its branches were further explored and denervated if significant EMG activity was still present.

### The efficacy evaluation criteria

The efficacy of treatment was evaluated by an independent physician who was not involved in the patient’s care or treatment plan. The doctor only assessed the degree of improvement in symptoms after surgery. The evaluation criteria are as follows: ① Cure: normal posture and activities of the head and neck were restored, and normal work, study and life were possible; ② Significant effect: the posture of the head and neck were roughly normal, and torticollis only occurred during exertion and stress; ③ Effect: the symptoms of torticollis were improved compared with those before surgery, and the angle and strength of obliquity were reduced; ④ No effect: there was no change compared with before surgery.

### Statistical analysis

Although there was no comparison of grouping and inter group differences, descriptive statistical (frequency and percentages) was used to analyze the data in this study.

## Results

Patients with cervical focal ST were included in the group, including 56 males and 40 females, with a mean age of 42 years, ranging from 18 to 72 years, and a 15-month-long mean course of the disease. There were 72 cases of rotational type, three cases of anterior flexion type, 10 cases of retroflexion type, and 11 cases of lateral bending type.

The distribution of spastic neck muscles of the four types of ST during involuntary movements was evaluated by EMG. Sternocleidomastoid muscle on the rotating side; In the lateral flexion type, the Grade III spastic muscles were the splenius capitis, levator scapulae, sternocleidomastoid and scalenus muscle. In the retroflexion type on the flexion side, the Grade III spastic muscles were the bilateral semispinalis and bilateral splenius capitis. In the anterior flexion type, the Grade III spastic muscles were the bilateral sternocleidomastoid. In the seven cases of rotational type, the spastic muscles were not only limited to the responsible muscles, but the main antagonist muscles were also obviously spastic (see Table 1).

**Table 1:** Distribution of spastic neck muscles of the four types of ST during involuntary movements (Grade III spasm/Grade II spasm/Grade I spasm

Type of muscle	Rotational type (n = 72)		Anterior flexion type (n = 11)		Retroflexion type (n = 10)		Lateral bending type (n = 3)	
	Rotating side Contralateral side		Flexion side	Contralateral side	Left	Right	Left	Right
splenius capitis	45/27/0	4/3/0	10/1/0	S	7/3/0	7/3/0	S	S
splenius cervicis	42/30/0	3/4/0	5/6/0	S	7/3/0	7/3/0	S	S
semispinalis capitis	30/42/0	2/5/0	0/7/4	S	5/5/0	5/5/0	S	S
semispinalis cervicis	35/37/0	2/5/0	0/6/5	S	4/6/0	4/6/0	S	S
Sternocleidomastoid	S	25/40/7	7/4/0	S	S	S	3/0/0	3/0/0
superior oblique muscle	S	S	S	S	0/5/4	0/5/4	S	S
inferior oblique muscle	S	S	S	S	0/6/3	0/6/3	S	S
rectus capitis posterior major	0/8/11	S	0/6/3	S	0/4/5	0/4/5	S	S
trapezius	0/16/10	S	0/4/1	S	0/1/0	0/1/0	S	S
levator scapulae	0/25/11	S	3/5/0	S	0/3/4	0/3/4	S	S
longissimus capitis	0/13/14	S	0/0/3	S	0/4/5	0/4/5	S	S
scalenus muscle	15/23/0	S	8/3/0	S	S	S	S	S

Note: S: electrical silence; in the rotational type, the Grade III spastic muscles were the splenius capitis and semispinalis and ramus and the contralateral

The postoperative electromyographic activity of the neck was recorded according to the EMG recording method used before the surgery. A satisfactory criterion of denervation was defined as a decrease of Grade III/II spasm to Grade I or electrostatic rest during involuntary movements in the responsible muscle. Before the surgery, 631 Grade II/III spastic muscles were recorded. Postoperatively, 599 met the denervation criteria, with an overall excellent rate of 95% (599/631). There were seven cases of preoperative antagonistic muscle spasm whose antagonistic muscles were not treated. Three spontaneously resolved spastic muscles after surgery, while the other four had no improvement (see Table 2).

**Table 2:** Postoperative efficacy evaluation

Overall excellent rate		
Grade II/III spastic muscles	Muscles met the denervation criteria postoperatively	Overall excellent rate
631	599	95%(599/631)
**Efficacy**		
	Number of patients / total number of patients	Ratio
Cure	64/96	66.6%
Significant effect	12/96	12.5%
Effect	14/96	14.5%
No effect	6/96	6.2%

In evaluating this group of cases, the cure category included 66.6% (64/96) of cases, significant effect included 12.5% (12/96), effect included 14.5% (14/96) and no effect included 6.2% (6/96). There was no death, disability or serious complications (see Table 2).

## Discussion

In our study, by analysing the preoperative EMG results, we determined that the responsible muscles for Grade III spasm are mainly located in the splenius capitis, splenius cervicis, semispinalis capitis, semispinalis cervicis, sternocleidomastoid, scalenus muscle and other long muscles, whose muscle fibres are wide. Some of them span several spinous processes and are prime mover muscles, which are the main responsible muscles. Longissimus capitis, superior obliquus, inferior obliquus, rectus capitis posterior major and other short posterior neck muscles are Grade II/I spasm-responsible muscles, which are thin and weak. Even if the spasm is strong, they cannot control the direction of the head and are followers. The rest of the muscles in between are the synergetic muscles. The synergetic and follower muscles form the secondary responsible muscles, and the distribution of the responsible muscles in this study was consistent with previous reports in the literature [9].

There are 27 pairs of neck muscles, 14 in the anterior neck and 13 in the posterior neck. In addition to the 12 pairs of muscles mentioned above, other muscles are involved in neck activities, such as the suprahyoid and infrahyoid muscles and the omohyoid and geniohyoid muscles involved in forward head flexion [9]. These deep muscles have short, small muscle fibres, making it difficult to accurately locate and puncture them to record electromyographic activity before surgery. They are innervated by the anterior branch of the spinal nerve, and denervation requires opening the canalis spinalis, which means greater trauma and related surgical complications. These short and small muscle fibres are less powerful and play a much smaller role than the muscles above in the development of the torticollis, and they are not treated in the ‘triple operation’ [10]. This part of the muscle and the residual spastic muscles after the ‘triple operation’ are less powerful. However, the antagonist muscles still need to be partially preserved to balance their power to restore the head and neck posture. Therefore, the function of the antagonist muscles is of great value in predicting postoperative efficacy. We rated the function of the major antagonist muscles preoperatively as excellent, good or poor according to EMG performance. In this study, the preoperative function of the antagonist muscles was rated as poor in all six cases assessed with no effect. In two of these cases, the antagonist muscle showed electrical silence during voluntary and involuntary movements before surgery, displaying ‘disuse atrophy’. In four cases, the antagonist muscle showed spasm before surgery. After postoperative denervation of the responsible muscle, the spasm of the antagonist muscle itself did not improve, nor did it improve the abnormal head position. This indicated that its spasm did not produce the force it should have when the muscle contracted; the muscle was in a ‘disuse state’ as in ‘disuse atrophy’, the mechanism of which needs further study.

During denervation, the nerves do not correspond to the spastic muscles one by one but present a variety of crossover; surgical treatment requires multiple complementary approaches to achieve highly selective spasm reduction. This study’s denervation rate was 95%, and the main responsible muscles had satisfactory denervation. After surgery, the residual spastic muscles were primarily the posterior cervical short muscles; this was related to the difficulty of complete denervation because the innervated nerve was the C1 nerve adjacent to the vertebral artery. The function of the antagonist muscles determines whether to ‘rebalance’. More clinical studies are needed to determine whether other surgical methods, including DBS, are a better choice in cases where preoperative electrophysiology showed the antagonist muscle had significant spasm or ‘disuse atrophy’ and the prognosis of ‘triple surgery’ was poor [11]. In our study, the primary and secondary roles of the responsible muscles were clarified by preoperative electrophysiology and denervated by intraoperative electrophysiology to minimise abnormal pulling forces.

- There were some limitations in this study. First, this study did not group patients and compare differences between groups, which weakened the reliability of the results. Second, the detailed assessment method was not a validated evaluation. It was necessary to assess clinical outcomes with a more diverse battery of outcomes in further trials. Third, based on practical reasons, the sample size of this study was small. There was a need for further research to explore this with a larger, more diverse cohort in a randomised controlled trial design.

## Conclusion

In summary, electrophysiological examination and intraoperative application may have a positive value in selecting the operative method, improving the rate of denervation and evaluating the prognosis of the ‘triple operation’.

## Data Availability

All data generated or analyzed during this study are included in this published article.
